# Linking kindling to increased glutamate release in the dentate gyrus of the hippocampus through the STXBP5/tomosyn‐1 gene

**DOI:** 10.1002/brb3.795

**Published:** 2017-08-13

**Authors:** Seth R. Batten, Elena A. Matveeva, Sidney W. Whiteheart, Thomas C. Vanaman, Greg A. Gerhardt, John T. Slevin

**Affiliations:** ^1^ Department of Psychology University of Kentucky College of Arts and Sciences Lexington KY USA; ^2^ Department of Molecular & Cellular Biochemistry University of Kentucky Medical Center Lexington KY USA; ^3^ Department of Neuroscience University of Kentucky Medical Center Lexington KY USA; ^4^ Neurology Service Veterans Affairs Medical Center Lexington KY USA; ^5^ Department of Neurology University of Kentucky Medical Center Lexington KY USA; ^6^ Department of Pharmacology and Nutritional Sciences University of Kentucky Medical Center Lexington KY USA

**Keywords:** epileptogenesis, microelectrode, SNAREs, SV2, synaptopathies

## Abstract

**Introduction:**

In kindling, repeated electrical stimulation of certain brain areas causes progressive and permanent intensification of epileptiform activity resulting in generalized seizures. We focused on the role(s) of glutamate and a negative regulator of glutamate release, STXBP5/tomosyn‐1, in kindling.

**Methods:**

Stimulating electrodes were implanted in the amygdala and progression to two successive Racine stage 5 seizures was measured in wild‐type and STXBP5/tomosyn‐1^−/−^ (Tom^−/−^) animals. Glutamate release measurements were performed in distinct brain regions using a glutamate‐selective microelectrode array (MEA).

**Results:**

Naïve Tom^−/−^ mice had significant increases in KCl‐evoked glutamate release compared to naïve wild type as measured by MEA of presynaptic release in the hippocampal dentate gyrus (DG). Kindling progression was considerably accelerated in Tom^−/−^ mice, requiring fewer stimuli to reach a fully kindled state. Following full kindling, MEA measurements of both kindled Tom^+/+^ and Tom^−/−^ mice showed significant increases in KCl‐evoked and spontaneous glutamate release in the DG, indicating a correlation with the fully kindled state independent of genotype. Resting glutamate levels in all hippocampal subregions were significantly lower in the kindled Tom^−/−^ mice, suggesting possible changes in basal control of glutamate circuitry in the kindled Tom^−/−^ mice.

**Conclusions:**

Our studies demonstrate that increased glutamate release in the hippocampal DG correlates with acceleration of the kindling process. Although STXBP5/tomosyn‐1 loss increased evoked glutamate release in naïve animals contributing to their prokindling phenotype, the kindling process can override any attenuating effect of STXBP5/tomosyn‐1. Loss of this “braking” effect of STXBP5/tomosyn‐1 on kindling progression may set in motion an alternative but ultimately equally ineffective compensatory response, detected here as reduced basal glutamate release.

## 1 INTRODUCTION

Kindling is a process whereby repeated electrical stimulation of certain brain areas, particularly the limbic system, with a fixed current strength and duration causes progressive and permanent intensification of epileptiform activity resulting in generalized seizures (Goddard, McIntyre, & Leech, 1969). Kindling in rodents has been well characterized both electrographically and behaviorally with progressive stages thought to mimic partial and secondary generalized human epilepsy (Racine, 1972). Once an animal reaches a fully kindled state, kindling‐evoked seizures readily occur for the rest of that animal's life, even after periods of abstinence (Bertram, 2007; Coulter, McIntyre, & Loscher, 2002; Racine, 1972). The persistence of seizure activity, even after electrical stimulations have ceased, is thought to derive from robust, permanent neurobiochemical changes in the brain; however, the exact mechanism is unknown (McIntyre & Gilby, 2006). Thus, understanding the molecular mechanism of the “kindling phenomenon” may help elucidate some of the molecular pathology of human epileptogenesis.

Synaptic glutamate release is an intricate and highly regulated process that includes several proteins, which facilitate synaptic vesicle docking, priming, and fusion with the plasma membrane (Südhof, 2013). The minimal protein requirements needed for neurotransmitter (NT) release are the plasma membrane‐bound t‐SNAREs, syntaxin and synaptosomal‐associated protein 25 (SNAP‐25), and the vesicle‐bound v‐SNARE, synaptobrevin/vesicle‐associated membrane protein 2 (VAMP‐2) (Jahn & Scheller, 2006). Neuronal depolarization leads to an increase in presynaptic intracellular Ca^2+^ that promotes membrane fusion and NT release. Membrane fusion requires the formation of a heterotrimer of these proteins, referred to as the 7‐Svedberg SNAP (soluble N‐ethylmaleimide‐sensitive factor [NSF] attachment protein) receptor (7S SNARE) complex (7SC) (Brunger, 2005; Jahn & Scheller, 2006; Weber et al., 1998).

Several proteins regulate the formation of the 7SC and thus NT release (Lonart & Südhof, 2000). These proteins include those that affect the dynamics of the “readily releasable pool” of NT‐containing vesicles, for example, Munc13s, Complexins, SV2s, and STXBP5/tomosyn‐1, as well as those that recycle SNARE complexes, for example, NSF and α‐SNAP (Ashery et al., 2000; Custer, Austin, Sullivan, & Bajjalieh, 2006; Hu et al., 2002; Whiteheart, Schraw, & Matveeva, 2001). STXBP5/tomosyn‐1 is thought to function as a negative regulator of SNARE‐mediated membrane fusion, primarily by sequestering the syntaxin‐1a and SNAP‐25 heterodimer and inhibiting 7SC formation (Ashery, Bielopolski, Barak, & Yizhar, 2009; Sakisaka et al., 2008; Willams et al., 2011). STXBP5/tomosyn‐1's C‐terminal VAMP‐like domain binds the t‐SNARE heterodimer and regulates access to v‐SNAREs. Two isoforms, tomosyn‐1 and tomosyn‐2, are preferentially expressed in the neuronal synapse (Yokoyama, Shirataki, Sakisaka, & Takai, 1999). STXBP5/tomosyn‐1 is highly expressed in glutamatergic synapses with differential expression within these synapses in the mouse hippocampus (Barak et al., 2010; Yokoyama et al., 1999). Several experimental models show that loss of STXBP5/tomosyn‐1 increases neurotransmitter release, excitatory postsynaptic potentials, and decreases odor memory (Chen, Richlitzki, Featherstone, Schwarzel, & Richmond, 2011; Dybbs, Ngai, & Kaplan, 2005; Gracheva et al., 2007; Sakisaka et al., 2008), consistent with STXBP5/tomosyn‐1 being a negative regulator of neurotransmitter release.

Given its role in neurotransmitter release, STXBP5/tomosyn‐1 is an ideal target to manipulate presynaptic glutamatergic neurotransmission to understand how glutamatergic neurotransmission contributes to kindling‐induced epilepsy. Here we report the use of glutamate biosensors with high temporal and spatial resolution to measure resting, spontaneous, and evoked glutamate from hippocampal subregions (dentate gyrus [DG], CA3, and CA1) in naïve and kindled wild‐type (Tom^+/+^) and knockout (Tom^−/−^) mice. Our results show that Tom^−/−^ mice have a kindling‐sensitive phenotype and that naïve Tom^−/−^ mice have higher KCl‐evoked glutamate release before kindling. However, the fully kindled state alone is associated with significantly enhanced KCl‐evoked and spontaneous glutamate release in the DG compared to all other brain regions in both kindled and naïve mice. Interestingly, basal glutamate levels were lower in kindled Tom^−/−^ mice suggesting a compensatory mechanism that may be triggered by kindling progression. These results suggest that a critical factor, possibly a driver, in the kindling phenomenon (and potentially in epileptogenesis) is the evolution of increased glutamate release in the hippocampal DG; however, once a fully kindled state is reached, STXBP5/tomosyn‐1 may not be important in modulating glutamate release and maintaining the kindled state.

## 2 MATERIALS AND METHODS

### 2.1 Preparation of kindled and control animals and tissues

#### 2.1.1 Animals

The STXBP5/Tom^−/−^ mice on a 50% 129Sv, 25% C57BL/6, and 25% DBA/2 background were generated as described previously (Sakisaka et al., 2008) and kindly provided by Dr. Yoshimi Takai (Department of Biochemistry and Molecular Biology, Kobe University Graduate School of Medicine, Kobe, Japan). All work with animals was done with approval from the University of Kentucky IACUC. Genotyping was performed by PCR using DNA prepared from tail biopsies (Sakisaka et al., 2008); wild‐type littermates were used as controls. These STXBP5/Tom^−/−^ mice were shown to be lacking tomosyn‐1, but none of the other elements of the secretory machinery by quantitative western blotting of different tissues (Sakisaka et al., 2008; Ye et al., 2014). For the kindling progression data, 14 Tom^+/+^ and 15 Tom^−/−^ mice were used. Six naïve Tom^+/+^ and five Tom^−/−^ mice were used in collection of the naïve glutamate data. For glutamate measurements in kindled mice, six fully kindled mice were used and were compared to the naïve mice above (*n* = 11) (see below and in figure captions for further details).

#### 2.1.2 Surgical preparation for kindling electrode

Surgery was performed in a David Kopf stereotaxic apparatus fitted with a mouse nose cone adapter. Mice were anesthetized and maintained with 2%–3% isoflurane during surgery. Teflon‐coated, stainless steel bipolar twisted electrodes were implanted in the right amygdala using coordinates from bregma (AP: −1.5; ML: +3.5; DV: −4.6) and adapted from Paxinos and Franklin (2003). Animals recovered for 10–14 days, postsurgery, prior to initiation of kindling. A random sampling from each group of treated animals was processed for histological analysis to assess electrode placement. Surgery specifics have been described previously (Matveeva, Vanaman, Whiteheart, & Slevin, 2007; Slevin & Ferrara, 1985).

#### 2.1.3 Kindling techniques

All kindling methods have been described previously (Matveeva, Whiteheart, & Slevin, 2003). Briefly, animals were tested for their after‐discharge (AD) threshold prior to kindling using a Grass S88 stimulator and an EEG. Once the AD was determined for each animal it was used to stimulate that respective animal once/day, 5 days/week. Full kindling for this study was operationally defined as a Racine (1972) stage 5 seizure, consisting of rearing and falling, occurring after administration of two consecutive stimuli. No animals experienced any behavioral signs of a seizure 1 week prior to analysis; this was determined by daily inspection of cages. However, split screen video electroencephalographic (SSV‐EEG) surveillance was not used; therefore, a seizure may have gone undetected. However, spontaneous seizures rarely occur unless the subject receives “extended kindling,” a process that typically involves hundreds of spaced electrical stimulations (Bertram, 2007). As the animals experienced only two stage 5 seizures, it is less likely than not that they experienced a spontaneous seizure. That said, the Tom^−/−^ mice were more easily kindled and thereby more likely to have spontaneous seizures, even after experiencing only two stage 5 seizures. Previously, we demonstrated that a generalized seizure per se does not affect the 7SC ratio in rats (Matveeva et al., 2003), thus even if a seizure occurred it is unlikely that it confounded our biochemical measurements.

#### 2.1.4 In vivo glutamate measurements

Enzyme‐based biosensors selective for glutamate were used to measure resting and evoked glutamate in vivo. Thirty days after experiencing two consecutive Racine stage 5 seizures, mice were anesthetized using isoflurane and glutamate measurements were collected using methods adapted from Matveeva et al. (2012); this was a nonsurvival surgery.

#### 2.1.5 Preparation of glutamate biosensors

Microelectrode arrays (MEAs) consisting of four platinum recording sites 20 μm × 150 μm arranged in dual pairs were adapted to selectively measure glutamate. A roughly 5 μl mixture of 1% bovine serum albumin (BSA), 0.125% glutaraldehyde, and 1% glutamate oxidase (GluOx) was manually placed (~0.1 μl drop) on the bottom two platinum recording sites using a gastight Hamilton^®^ syringe. Glutamate oxidase (GluOx) breaks down glutamate into α‐ketoglutarate and the recorder molecule, H_2_O_2_, which is oxidized at the electrode surface. The same procedure was used to apply 1% BSA and 0.125% glutaraldehyde on the top two sites of the electrode, which allows for self‐referencing as described previously (Burmeister & Gerhardt, 2001; Day, Pomerleau, Burmeister, Huettl, & Gerhardt, 2006). After the MEAs were coated, they were cured for >72 hr and then plated with m‐phenylenediamine (mPD), a size exclusion layer, to allow for more selective glutamate measurements. The mPD (5 mmol/L) solution was made in deoxygenated phosphate‐buffered saline (PBS). The MEA was then connected to the FAST 16 MKII potentiostat system (Fast Analytical Sensing Technology Mark II, Quanteon, LLC) and the four platinum sites of the electrode were submerged in the mPD solution. The platinum recording sites of the electrode were plated with mPD for 10–20 min via the electroplating tool built into the software and they were allowed to sit for >24 hr before use.

#### 2.1.6 MEA placement and in vivo recordings under anesthesia

All mice were anesthetized using 1%–3% isoflurane, placed in a stereotaxic frame (David Kopf Instruments, Tujunga, CA), and a bilateral craniotomy was performed exposing both overlying hemispheres of the bilateral hippocampi. For kindled mice, the kindling head cap was removed once the mice were placed in the stereotaxic frame, then the bilateral craniotomy was performed. Body temperature was maintained at 37°C using a circulating water bath attached to a water pad (Gaymar Industries, Orchard Park, NY). A small burr hole was made anterior to the site of the craniotomy and a small Ag/AgCl reference electrode was implanted into the brain. Glutamate measurements for resting, spontaneous, and potassium‐evoked release of glutamate were recorded from discrete subregions of the hippocampus including the DG (AP: −2.3; ML: ±1.5; DV: −2.1), CA3 (AP: −2.3; ML: ±2.7; DV: −2.25), and CA1 (AP: −2.3; ML: ±1.7; DV: −1.4). Anterior–posterior and medial–lateral coordinates for MEA implantation were calculated relative to bregma and dorsal–ventral coordinates were taken from the surface of the brain (Paxinos & Franklin, 2003). A prepulled single barrel glass micropipette (inner diameter ~10 μm; A‐M Systems Inc., Everett, WA) was used to locally apply KCl in distinct hippocampal subregions to measure stimulus‐evoked glutamate release. The micropipette was positioned in the center of the four pairs of platinum recording sites at a height between 50–100 μm, and attached to the electrode using Sticky Wax (Kerr Lab Corporation, Orange, CA). The micropipette attached to the MEA was then filled with filtered 70 mmol/L KCl (70 mmol/L KCl, 79 mmol/L NaCl, 2.5 mmol/L CaCl_2_; pH 7.4) and the MEA was attached to the stereotaxic frame. The micropipette was then attached to a Picospritzer III (Parker Hannifin Corporation, NJ) via rubber tubing to control the volume of KCl ejected into each subregion of the hippocampus. A stereomicroscope, equipped with a reticule, was used to monitor the volume of KCl ejected (Friedemann & Gerhardt, 1992). Note that DG, CA3, and CA1 glutamate measurements were taken from all animals in both brain hemispheres and electrode placement for all brain regions were counterbalanced, thus there should be no order effects in our measurements. Furthermore, both basal glutamate and spontaneous release measures were taken before KCl was ejected in each brain region, thus KCl application did not affect these measurements. It is also worth noting that basal measurements and spontaneous measurements are functionally distinct: basal measurements are true resting levels, whereas spontaneous measures are glutamate release events that occur during no KCl stimulation.

**Figure 1:**
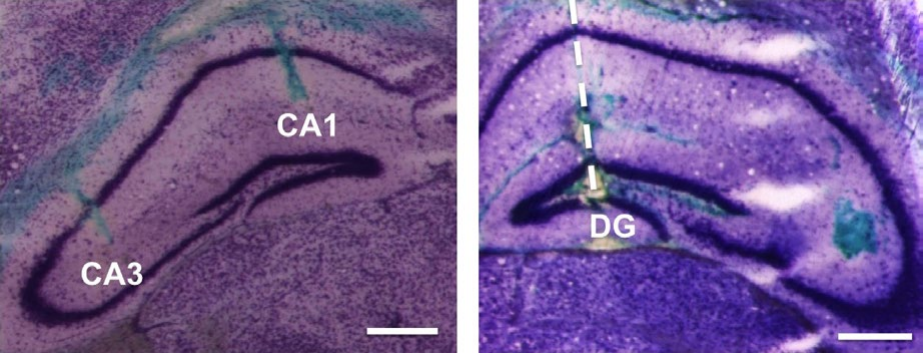
Representative histology of glutamate biosensor placement in mouse hippocampal subregions. After completion of electrochemical recordings green ink was ejected into each hippocampal subregion where recordings occurred to confirm electrode placement. Shown are micrographs of 40 μm coronal sections stained with Cresyl violet. Left figure: Dorsal–ventral placement of the electrode for glutamate measurements in the CA3 and CA1. Right figure: Dorsal–ventral placement of the electrode for glutamate measurements in the DG. Scale bar = 500 μm

Electrode placement was assessed after the MEA recordings were completed. Specifically, the micropipette was filled with green ink (Special Green Ink; KOH‐I‐NOOR Co.), placed back in the hippocampal subregions where glutamate recordings were done, and the ink was locally ejected. After MEA recordings, animals were euthanatized and brains were removed and flash frozen; 40 μm slices were obtained using a cryostat. The slices were stained using Cresyl violet (Sigma‐Aldrich) and were imaged to confirm MEA placement (Figure 1 left and right).

### 2.2 Data analyses

#### 2.2.1 Kindling and glutamate analysis

After‐discharge thresholds and number of stimulations for kindled Tom^+/+^ and Tom^−/−^ mice were each analyzed by the Mann–Whitney *U* test because the data were not normally distributed and violated homogeneity of variance. The ipsilateral/contralateral 7SC band intensity ratios of kindled mice were analyzed using the Mann–Whitney *U* test because the data were not normally distributed and violated homogeneity of variance. The quantified secretory machinery proteins from kindled hippocampi were analyzed by ANOVA. The amperometric data collected in naïve and kindled Tom^+/+^ and Tom^−/−^ mice were analyzed using custom exported Excel™‐based software (Quanteon LLC, Lexington, KY). The parameters were resting (μmol/L), spontaneous (mol/L), and KCl‐evoked glutamate release (mol/L), as well as the time to rise of the evoked glutamate peak (*T*_rise_; s), the time to 80% decay of the evoked glutamate peak (*T*_80_; s), the slope of the linear regression of the natural log transformation of the decay over time (*k*_−1_; s^−1^), and the peak area under the evoked glutamate peak (area under the curve [AU]) (Hascup et al., 2007; Hinzman et al., 2010; Matveeva et al., 2012). For resting levels and spontaneous release events, averages were taken after the electrode reached a stable baseline for at least 30 min and before any KCl was used to stimulate the system in a given brain area. After resting data were collected, 100 nl of 70 mmol/L KCl was ejected in each brain area to evoke glutamate release; the peak amplitude concentration of glutamate release as well at the *T*_rise_, *T*_80_, *k*_−1_, and peak area measurements in each brain area is an average of three evoked peaks. For all glutamate measurements the background current from the sentinel sites was subtracted from the current measured at the GluOX‐coated sites for a more accurate glutamate measure in micromolar concentrations. The differences in naïve Tom^+/+^ and naïve Tom^−/−^ glutamate release were analyzed using a two‐way mixed ANOVA. Analysis of the relationships between genotype, kindling, brain area, and/or hemisphere was analyzed using an omnibus (four‐way) ANOVA. Where appropriate, a Bonferroni correction post hoc analysis was used to probe interactions. Statistical significance was determined as *p *<* *.05 for all analyses. Note that no genotype effects were seen in kindled animals for the glutamate measurements. Thus, all glutamate measurements are collapsed across genotype, hence the kindled glutamate figures only show comparisons between naïve and kindled animals with no regard to genotype.

#### 2.2.2 Analysis of 7S SNARE complexes (7SC) and SNARE regulators

All animals were analyzed approximately 30 days after experiencing two Racine stage 5 seizures. For some, tissue was harvested after MEA measurements. Animals were euthanatized by decapitation and their brains were removed and the hippocampi excised on ice. Percoll gradient‐purified synaptosomes were prepared from individual hippocampi as previously described (Dunkley, Jarvie, Heath, Kidd, & Rostas, 1986; Dunkley et al., 1988). Synaptosome extracts were prepared by incubating equal quantities of synaptosomal protein in sodium dodecyl sulfate polyacrylamide gel electrophoresis (SDS‐PAGE) sample buffer for 30 min at 37°C; under these conditions monomeric SNAREs are denatured, but the 7SC is thermally stable and fails to disassemble (Matveeva et al., 2003). Upon completion of electrophoresis and transfer to PVDF membranes (Millipore), these complexes were probed by western blotting using antibodies against the t‐SNARE, syntaxin 1 (HCP‐1; Inoue & Akagawa, 1992) as described previously (Matveeva et al., 2003). Detection of 7SC was performed using alkaline phosphatase‐coupled secondary antibodies with Vistra ECF™ for visualization and images were obtained using a Typhoon 9400 imager (GE Healthcare Bio‐Sciences, Piscataway, NJ). The raw data collected were the integrated fluorescence intensities for all pixels in a given protein band as determined by ImageQuant 5.2 software (GE Healthcare Bio‐Sciences, Piscataway, NJ) in arbitrary units. To normalize for protein loading the fluorescence intensity of all 7SC bands was standardized to the intensity of the syntaxin‐1 monomer as in Matveeva et al. (2011). The 7SC complex was then analyzed by comparing the relative ratio of protein from the ipsilateral hippocampus compared to the contralateral hippocampus (ipsilateral/contralateral) for both kindled genotypes using the following equation: [7SC ipsilateral/syntaxin‐1]/[7SC contralateral/syntaxin‐1].

Other secretory machinery proteins were analyzed as above. NSF was detected with the 2E5 monoclonal antibody (Tagaya, Wilson, Brunner, Arango, & Rothman, 1993; Whiteheart et al., 1994). The monoclonal antibody to SV2 (which detects both A and B isoforms) was obtained from the Developmental Studies Hybridoma (Iowa City, IA). Fluorescence intensities of the bands in each lane were normalized to the intensity of the syntaxin‐1 band in the same lane. To determine “protein laterality” (relative amount of protein in ipsilateral vs. contralateral hippocampus), the following equation was used: [(contralateral/syntaxin‐1 + ipsilateral/syntaxin‐1)/2]/(mean contralateral control/syntaxin‐1). If no changes in protein level occur, then the ratio should be one. Note these methods are based on the previously published work (Matveeva et al., 2007).

## 3 RESULTS

### 3.1 Effect of STXBP5/tomosyn‐1 on kindling

**Figure 2:**
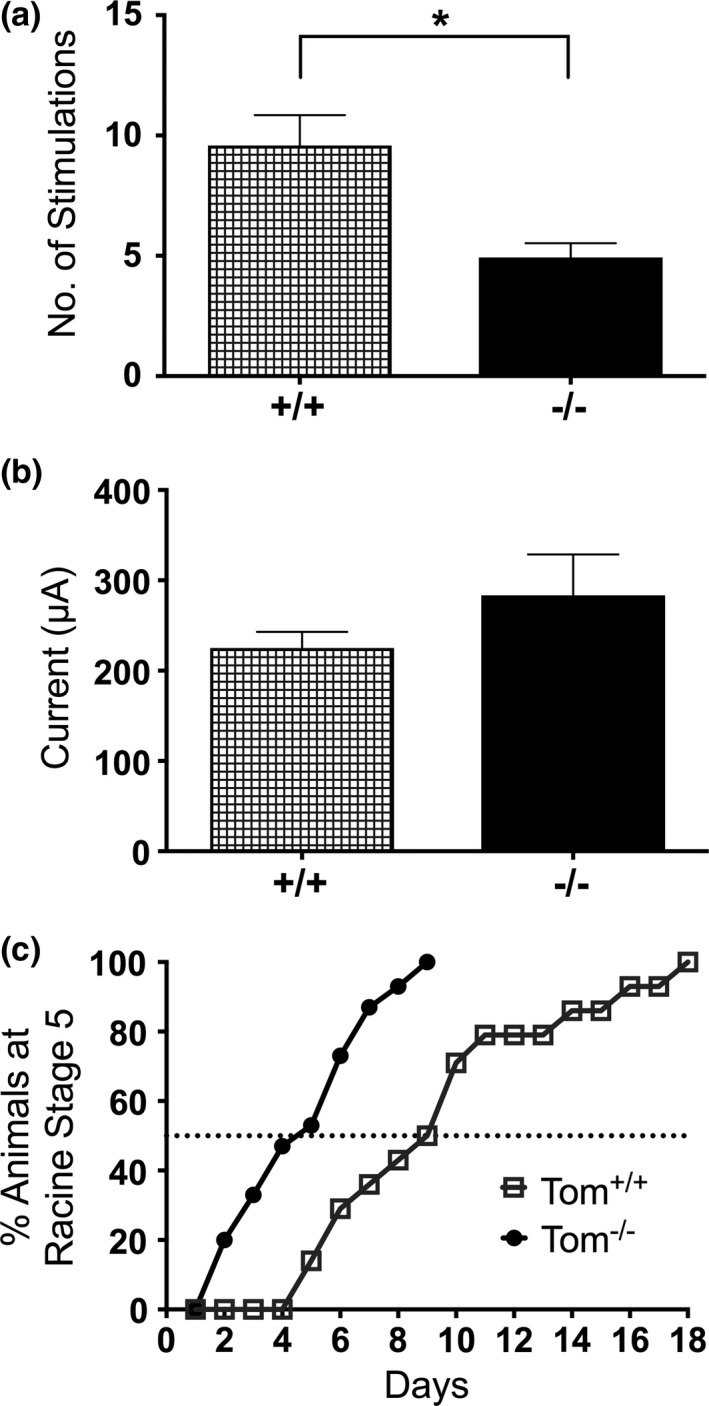
Mice lacking STXBP5/tomosyn‐1 kindle faster than wild‐type mice. (a) The average number stimulations (once per day) required to obtain a stage 5 seizure on 2 consecutive days. Data are presented as mean ± *SEM*;* n* = 14 for Tom^+/+^ mice and *n* = 15 for Tom^−/−^ mice [*U *=* *31, *p =* *.0028, Mann–Whitney *U* test). (b) The average current required to produce an AD for Tom^+/+^ versus Tom^−/−^ mice (*U *=* *81, *p *=* *.67, Mann–Whitney *U* test). (c) Time course to cumulatively reach a Racine stage 5 seizure (1 stimulation per day) for Tom^+/+^ (grey line, *n* = 14) and Tom^−/−^ (black line, *n* = 15) mice. Note the 50% line corresponds to the average number of stimulations seen for each genotype in (a)

Considering STXBP5/tomosyn‐1's role as a negative regulator of glutamate release, we chose to first assess if Tom^−/−^ mice had a kindling‐sensitive phenotype, which would suggest that dysregulation of glutamate release contributes to epileptogenesis. Kindling was induced much more rapidly in Tom^−/−^ mice compared to controls. Fewer stimulations were required to reach a fully kindled state in Tom^−/−^ animals than in control mice (Figure 2a). However, there were no significant differences in the AD threshold current between the two genotypes (Figure 2b). In addition to association with an initial inhibition of kindling onset, STXBP5/tomosyn‐1 appears to retard the development of kindling as depicted in Figure 2c, where the slopes of kindling evolution between the populations of Tom^+/+^ and Tom^−/−^ mice are different.

### 3.2 Alterations in the secretory machinery in kindled mice

To determine if the biochemical changes we previously identified (Matveeva, Vanaman, Whiteheart, & Slevin, 2008; Matveeva et al., 2003, 2007, 2011, 2012) were occurring in this model, we assessed the 7SC complex formation and the levels of specific proteins (SV2; NSF) 1 month following full kindling as operationally defined. Table 1 shows protein analysis of various components of the neurosecretory machinery from both kindled Tom^+/+^ and Tom^−/−^ mice. For a given protein determination, prior MEA measurements in animals of a specific genotype made no difference; hence, the values in Table 1 are from animals ± MEA measurements. As seen previously, there was an ipsilateral increase in 7SC, but there was no significant genotype‐specific difference (note that both ratios are above one in both genotypes suggesting only a kindling effect). Similarly, total levels of SV2 increased and NSF decreased in both kindled Tom^+/+^ and Tom^−/−^ mice, as previously reported for other kindling models (Matveeva et al., 2007; Ohno et al., 2009). There was no significant genotype‐specific difference as seen by the fact the NSF and SV2 levels for both genotypes are below one and above one, respectively, again suggesting only a kindling effect.

**Table 1:** Whole hippocampal 7SC and selected regulatory exocytic protein measures in kindled Tom^+/+^ and Tom^−/−^ mice (mean ± *SEM*)

	Kindled Tom^+/+^ (*n*)	Kindled Tom^−/−^ (*n*)
7 SC^a^	1.3 ± 0.16 (8)	1.04 ± 0.13 (11)
NSF^b^	0.81 ± 0.35 (3)	0.95 ± 0.23 (5)
SV2^b^	1.22 ± 0.13 (7)	1.21 ± 0.12 (6)

a Ratio = [7SC ipsilateral/syntaxin 1]/[7SC contralateral/syntaxin 1], where unity is expected.

b Total SV2 protein = [(contralateral/syntaxin 1 +  ipsilateral/syntaxin 1)/2]/(mean contralateral control/syntaxin 1)]. If no changes in protein level occur, then the ratio should be unity.

### 3.3 Functional correlates of kindling, STXBP5/tomosyn‐1 deficiency, and glutamate dynamics

The data above suggested that STXBP5/tomosyn‐1 may act to mitigate kindling considering mice lacking STXBP5/tomosyn‐1 express a kindling‐sensitive phenotype. Our group and others previously showed that glutamate release dynamics are changed in kindled rats (Matveeva et al., 2012; Sun et al., 2013), thus it is possible that the kindling‐sensitive phenotype seen in Tom^−/−^ animals is, in part, due to glutamate system dysregulation through alterations in glutamate release. We assessed whether STXBP5/tomosyn‐1 loss and kindling affected glutamate neurotransmission in distinct hippocampal subregions in vivo in Tom^+/+^ and Tom^−/−^ mice by measuring extracellular resting glutamate, spontaneous and KCl‐evoked glutamate release, and the *T*_rise_, *T*_80_, *k*_−1_, and peak area of the evoked peak (Hascup et al., 2007, 2011; Hinzman et al., 2010; Matveeva et al., 2011, 2012).

**Figure 3:**
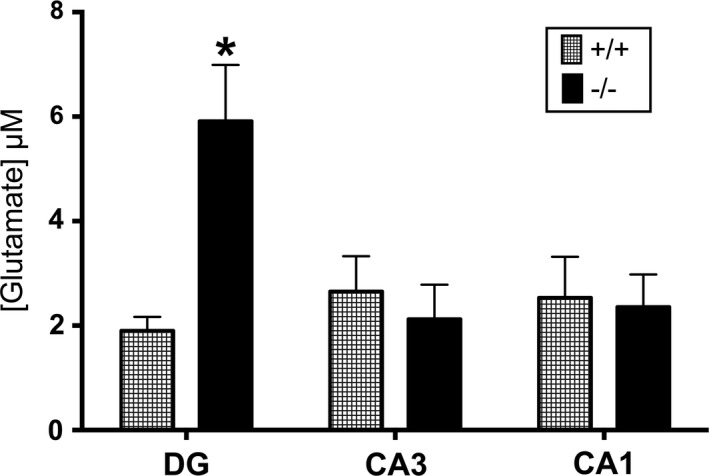
KCl‐evoked glutamate release in the hippocampus of naïve wild‐type and STXBP5/tomosyn‐1 null mice. All measurements are presented as mean ± *SEM* and were collected in naïve Tom^+/+^ (*n* = 6) and Tom^−/−^ (*n* = 5) mice. KCl‐evoked glutamate release is higher in the DG of naïve Tom^−/−^ mice (black bars) compared to the DG in Tom^+/+^ mice (hatched bars). There were no difference in CA3 or CA1 glutamate release between the two genotypes [*F*(2, 18) = 7.1, *p =* *.0054; Bonferroni correction]

Consistent with STXBP5/tomosyn‐1's role as a negative regulator of NT release, the KCl‐evoked glutamate release was greater in the DG of naïve Tom^−/−^ mice compared to naïve Tom^+/+^ mice (Figure 3). Other hippocampal regions showed no genotype‐dependent differences in KCl‐evoked glutamate release. There were also no differences in basal, spontaneous release, *T*_rise_, *T*_80_, *k*_−1_, or peak area measures in naïve mice (Table 2).

**Table 2:** Average glutamate measures from the whole hippocampus in naïve and kindled mice for both genotypes

Animal	DG	CA3	CA1	DG	CA3	CA1	DG	CA3	CA1
Average glutamate measures from the whole hippocampus in naïve and kindled Tom^+/+^ mice (mean ± *SEM*)									
	Tonic			*T*_rise_ (s)			*T*_80_ (s)		
Naïve Tom^+/+^	1.92 ± 0.52	1.55 ± 0.23	1.61 ± 0.25	2.07 ± 0.43	2.97 ± 0.60	2.99 ± 0.55	5.28 ± 1.15	3.65 ± 0.80	7.76 ± 0.96
Kindled Tom^+/+^	4.79 ± 2.83	3.09 ± 0.68	4.91 ± 2.66	3.69 ± 1.08	2.25 ± 0.60	3.80 ± 1.16	8.26 ± 2.18	8.42 ± 2.49	5.95 ± 0.86
	*K*_−1_ (s^−1^)			Peak area (AU)			KCl evoked (μmol/L)		
Naïve Tom^+/+^	0.96 ± 0.48	0.99 ± 0.29	0.51 ± 0.34	8.33 ± 2.85	10.21 ± 4.64	15.62 ± 5.38	1.90 ± 0.30	2.66 ± 0.50	2.54 ± 0.82
Kindled Tom^+/+^	0.20 ± 0.07	0.21 ± 0.09	0.16 ± 0.05	135.51 ± 82.04	70.11 ± 24.88	57.22 ± 23.20	34.48 ± 21.68	21.80 ± 10.61	18.56 ± 9.31
Average glutamate measures from the whole hippocampus in naïve and kindled Tom^−/−^ mice (mean ± *SEM*)									
	Tonic			*T*_rise_ (s)			*T*_80_ (s)		
Naïve Tom^−/−^	1.87 ± 0.34	2.10 ± 0.41	1.83 ± 0.33	2.72 ± 0.35	2.55 ± 0.48	1.95 ± 0.36	5.73 ± 0.98	4.21 ± 0.87	5.08 ± 1.23
Kindled Tom^−/−^	0.44 ± 0.08	0.94 ± 0.57	0.32 ± 0.05	1.76 ± 0.21	3.33 ± 1.31	1.41 ± 0.22	4.63 ± 1.52	7.77 ± 2.76	8.68 ± 2.59
	*K*_−1_ (s^−1^)			Peak area (AU)			KCl evoked (μmol/L)		
Naïve Tom^−/−^	0.31 ± 0.10	0.37 ± 0.13	0.59 ± 0.30	25.14 ± 7.39	9.27 ± 4.86	6.58 ± 1.47	5.91 ± 1.20	2.12 ± 0.50	2.36 ± 0.54
Kindled Tom^−/−^	0.51 ± 0.10	0.32 ± 0.12	0.54 ± 0.27	80.35 ± 33.11	46.94 ± 7.69	83.64 ± 35.39	23.65 ± 5.97	13.61 ± 4.32	11.91 ± 3.86

**Figure 4:**
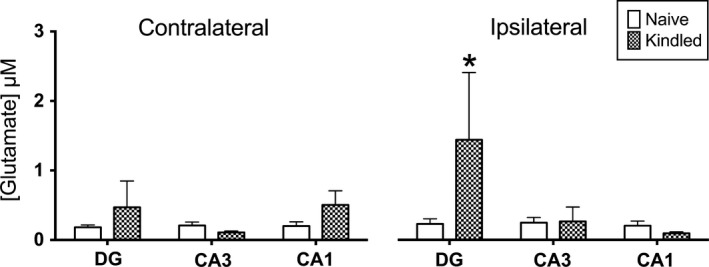
Spontaneous glutamate release is greater in the ipsilateral dentate gyrus of kindled mice. All measurements are presented as mean ± *SEM* and were collected from both naïve (*n* = 11) and kindled (*n* = 6) mice. These composite data show that the amplitude of spontaneous glutamate release is significantly higher in the ipsilateral DG of kindled animals compared to all other ipsilateral and contralateral brain regions in naïve (white bars) and kindled (checkered bars) animals except the contralateral kindled CA1 [*F*(2, 26) = 6.9, *p =* *.004; Bonferroni correction]

Loss of SXTBP5/tomosyn‐1 did not affect the amplitudes of spontaneous, transient glutamate release; however, kindling did have an effect, regardless of genotype (Figure 4). There were significantly larger spontaneous peaks in the DG ipsilateral to the kindling electrode compared to all other brain regions except for the kindled, contralateral CA1. We previously observed similar spontaneous changes in kindled rats (Matveeva et al., 2011), indicating that heightened spontaneous synaptic glutamatergic activity in DG correlates with kindling in rodents and suggests it may be universal across species.

**Figure 5:**
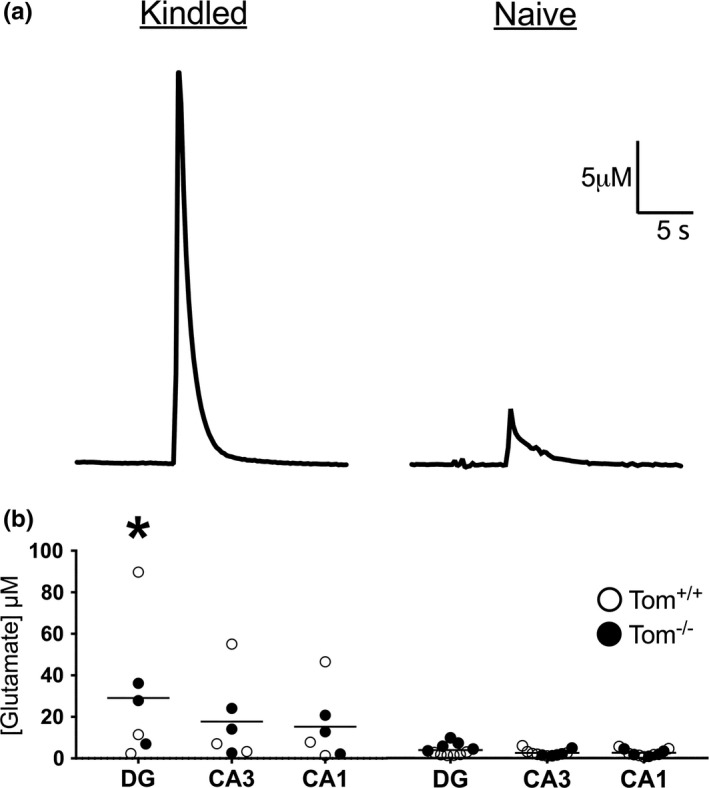
KCl‐evoked glutamate release is greater in the DG of kindled mice. All measurements as scatter plots with the mean indicated as a line were collected in naïve (*n* = 11) and kindled (*n* = 6) mice. (a) A representative trace from the right DG of kindled (left) and naïve (right) mice for KCl‐evoked glutamate release. (b) The amplitude of KCl‐evoked glutamate release is higher in the DG of kindled animals (left) compared to the DG, CA3, and CA1 in naïve animals (right) and the CA3 and CA1 of kindled animals [*F*(2, 26) = 6.0, *p =* *.0073; Bonferroni correction]. The white circles represent Tom^+/+^ mice and the black circles represent Tom^−/−^ mice. Notice how in the kindled animals there is no obvious trend for a genotype effect but there is an overall trend for a kindling effect

There was a genotype‐independent increase in the amplitude of KCl‐evoked glutamate release in the DG (both hemispheres) of kindled animals compared to kindled and nonkindled animals across all hippocampal subregions (Figure 5a and b; average data collapsed across hemispheres due to the lack of a hemisphere effect). Analysis of *T*_rise_, *T*_80_, *k*_−1_, and peak area of the evoked glutamate peaks showed no significant differences, suggesting no change in release mechanism, just the quanta of release events (Table 2). Interestingly, resting levels of glutamate were significantly reduced in kindled Tom^−/−^ mice compared to kindled Tom^+/+^ mice suggesting that there is a decrease in the basal extracellular concentration of glutamate released in the hippocampus of kindled Tom^−/−^ mice (Table 3).

**Table 3:** Average resting glutamate levels for kindled Tom^+/+^ and Tom^−/−^ mice in the whole hippocampus (mean ± *SEM*)

Kindling status	Tom^+/+^ resting (μmol/L)	Tom^−/−^ resting (μmol/L)
Naïve	1.7 ± 0.20	1.9 ± 0.20
Kindled	4.3 ± 1.3	0.57 ± 0.19^a^

a *p *<* *.05.

## 4 DISCUSSION

The most widely used models of epileptogenesis include electrical kindling, poststatus epilepticus (SE; chemical kindling with, e.g., kainate or pilocarpine) models of temporal lobe epilepsy (TLE), and models of traumatic brain injury‐induced epilepsy (Kharatishvili & Pitkanen, 2010; Loscher & Brandt, 2010; Stables et al., 2002). Post‐SE chemical kindling models, in which the acute triggering process of SE is frequently followed by a latency period with subsequent development of spontaneous motor seizures, closely mimic the clinical manifestations of human TLE and electrical kindling. Recordings from intracerebral implanted electrodes demonstrate that the first electrographic abnormalities in TLE without mesial temporal sclerosis (MTS) often appear within hippocampus (Van Roost, Solymosi, Schramm, van Oosterwyck, & Elger, 1998). This suggests that network changes in the hippocampal formation are important in epileptogenesis, but that morphological changes such as MTS while perhaps augmenting the process are not essential (Thom, 2014; Wang, Smith, Murphy, & Cook, 2010). A major drawback to all post‐SE chemical kindling models is that the epileptogenesis associated with most TLE is not initiated by an episode of status epilepticus, raising the question: what changes are primary to epileptogenesis and what are epiphenomena to the model? The intent of this study was to evaluate alterations in potential hippocampal network changes occurring at the level of synaptic neurotransmission. We chose to perform electrical kindling, operationally defining full kindling as two stage 5 seizures, to create a state where network change has occurred without gross morphological or inflammatory changes. In this regard, Osawa, Uemura, Kimura, and Sato (2001) have demonstrated that in the case of amygdalar stimulation, kindling develops without evidence of mossy fiber sprouting until after repeated kindled convulsions, following death of granule cells in the DG. In their study, animals experienced 29 ADs and two stage 5 kindled convulsions before these histopathological changes were seen. Amygdala kindled Tom^−/−^ mice in this study experienced an average of five ADs (one animal had 9) and two kindled convulsions. Tom^+/+^ mice had an average of 10 ADs (one animal had 18) and two kindled convulsions. The literature would suggest that neither genotype, but in particular the Tom^−/−^ mice, experienced a sufficient number of ADs and kindled convulsions to have undergone significant morphologic changes including neurodegeneration, inflammation, and mossy fiber sprouting.

The most striking effect seen in this study was the kindling‐sensitive phenotype of Tom^−/−^ mice. Significantly fewer stimuli were needed to reach a fully kindled state in Tom^−/−^ mice compared to controls. Since the AD currents were not different, it seems credible that Tom^−/−^ mice have a greater response to the same level of stimulation. In concert with this observation, the MEA measurements of naïve animals showed that KCl‐evoked glutamate release was increased in Tom^−/−^ mice, therefore it is plausible that each stimulation could result in higher glutamate release, which could enhance the kindling process. This is perhaps not surprising since our previous studies with VAMP‐2 heterozygous mice showed a delay in kindling and a decrease in glutamate release (Matveeva et al., 2012). Thus, our data imply that the dysregulation of glutamate release, whether increased or decreased, affects the kindling process.

It was expected that loss of STXBP5/tomosyn‐1 would also have increased glutamate release in kindled mice; however, this was not observed. Once animals were kindled, loss of STXBP5/tomosyn‐1 had no effect. There are several potential explanations for this result. First, it is possible that the fully kindled state represents a maximized state for glutamate release that cannot be exceeded. Thus, the increased glutamate release measured in naïve Tom^−/−^ mice cannot be observed in kindled animals because their release mechanisms are saturated as a result of kindling. An alternative explanation may relate to the small sample size of kindled animals (*n* = 3/genotype). It is possible that our lack of data supporting any genotype effect could be due to a type II error caused by the small sample size. For our analysis of the effect of kindling, all genotypes are collapsed to a population size of *n* = 6; thus, the observed differences are more likely real and not a type I error since this aggregate sample size and the results are similar to findings of previous studies (Matveeva et al., 2012; Rüden, Jafari, Bogdanovic, Wotjak, & Potschka, 2015).

Kindling has been shown to increase blood pressure and cardiac arrhythmias thus making kindled animals prone to death (Goodman, Homan, & Crawford, 1990). Tom^−/−^ mice, especially after kindling, were more sensitive to the manipulations of our experiments. Although we did not have 24 hr video surveillance, we found many Tom^−/−^ mice dead in a messy cage with strewn feces and the animals themselves with dried saliva and blood on their muzzles, strongly suggesting seizures as a cause of death and the consideration of a murine equivalent of SUDEP (sudden unexpected death in epilepsy). Some animals died during kindling provoked seizures. In our study of VAMP2 mutants (Matveeva et al., 2012) that were kindling resistant, we did not observe this high level of mortality associated with the kindling stimulation, the 30‐day postkindling period, or during subsequent MEA measurements. In total, 67% of the kindled Tom^−/−^ mice and 62.5% of the kindled Tom^+/+^ mice died during these studies. Another potential reason for the difference in mortality between the VAMP2 and STXBP5/tomosyn‐1 animals may relate to background, since the VAMP‐2 mice were on a pure C57B/6 genetic background. As in the previous studies by Sakisaka et al., 2008, we used null animals and littermate controls on a hybrid genetic background of C57B/6 and BDF1.

The only genotype effect seen in this study was in the low basal glutamate levels seen in the kindled Tom^−/−^ mice. This effect could be a compensatory response acting through presynaptic mGluR_2/3_ autoreceptors (Rohde et al., 2012; Watanabe et al., 2011). In support of this explanation, we previously showed that resting glutamate levels are modulated with an mGluR_2/3_ agonist and antagonist in freely moving animals (Hascup et al., 2010). Alternatively, there is also evidence that kindling may increase neuropeptide Y which has been shown to decrease resting glutamate levels in the hippocampus (Botterill, Guskjolen, Marks, Caruncho, & Kalynchuk, 2015; Klapstein & Colmers, 1997; Patrylo, van den Pol, Specer, & Williamson, 1999). Furthermore, data suggest that the NPY Y2 receptor is elevated in bilateral hilus and the NPY Y1 receptor is downregulated in DG 30 days following three stage 5 seizures of hippocampal‐kindled rats (Gobbi et al., 1998). Other data indicate mice with NPY gene deletion or inactivation show enhanced susceptibility to seizures, while endogenous overexpression of NPY protects from seizures and delays kindling epileptogenesis (Richihi et al., 2005). Thus, complicated GABA‐ and glutamatergic changes occur during kindling epileptogenesis that do not offer a ready explanation of why basal levels of glutamate are less in kindled Tom^−/−^ than kindled Tom^+/+^mice. That the diminution effect is only seen in kindled Tom^−/−^ animals may be due to an interaction between their higher evoked glutamate release in the naïve state and repeated kindling stimulations. Considering the small sample sizes, these effects should be considered cautiously and more credence should be given to the kindling findings that were independent of a genotype.

Further comment should be made regarding potential GABAergic mechanisms contributing to rapid epileptogenesis in the Tom^−/−^ mice. Bragin, Azizyan, Almajano, Wilson, and Engel (2005) have shown low‐voltage fast‐onset (LVF) and hypersynchronous‐onset (HYP) spikes preceding spontaneous seizures in freely moving rats that had SE‐induced kainic acid hippocampal injections. Levesque, Salami, Gotman, and Avoli (2012) demonstrated LVF and HYP in the rat pilocarpine model of TLE. Avoli and colleagues have recently studied the equivalent of LVF and HYP seizures in an in vitro slice preparation of rat perirhinal cortex (Köhling et al., 2016). They concluded their data best explained HYP seizures as a dynamic weakening of GABA_A_ receptor signaling. The artifact induced by the electrical kindling stimulus in our study obscures the initial components of the EEG trace making this model not amenable to studying LVP and HYP, including a potential GABAergic mechanism, in the transition from the preictal to the ictal state.

STXBP5/tomosyn‐1 may be most important at regulating glutamate early in kindling and epileptogenesis (Matveeva et al., 2008); thus, STXBP5/tomosyn‐1's absence may contribute to the faster progression we observed as well as to the increased evoked glutamate release seen in the naïve Tom^−/−^ mice before kindling. Once an animal is fully kindled, the effect of STXBP5/tomosyn‐1 is not apparent. It is formally possible that tomosyn‐2 compensates for the loss of STXBP5/tomosyn‐1 in the fully kindled state. We did not measure its levels. However, tomosyn‐2 is found postsynaptically suggesting that it may play a role in synaptic plasticity independent of regulating neurotransmitter release (Barak et al., 2010).

Others have shown that kindling increases glutamate release in the rat hippocampus (Geula, Jarvie, Logan, & Slevin, 1988; Jarvie, Logan, Geula, & Slevin, 1990; Minamoto et al., 1992). Here we show an increase in evoked and spontaneous glutamate release in the DG of the hippocampus in kindled mice. We have previously reported changes in DG glutamate dynamics in kindled animals (Matveeva et al., 2011, 2012). In fact, others have also found changes in the DG of the hippocampus using chemical and electrical kindling models (Popova, Aniol, Lazareva, Yu, & Gulyaeva, 2013; Sun et al., 2013). The DG is considered the “gatekeeper” of the trisynaptic glutamatergic throughput of the hippocampus (Tamminga, Southcott, Sacco, Wagner, & Ghose, 2012). The DG also has a high rate of neurogenesis (Aimone, Deng, & Gage, 2011). These factors may make the DG more susceptible to insults. Also, compensatory responses may occur downstream in CA3 and CA1 that may attenuate glutamate release; this could explain why no changes in glutamate release were observed in these areas. This is consistent with the spontaneous glutamate release dynamics seen in the rat hippocampus we previously reported (Matveeva et al., 2012). Thus, the current research adds to the preexisting body of work suggesting that glutamate dysregulation in the DG is a primary driver of epileptogenesis. Even more so, this research highlights the fact that though proteins involved in regulating glutamate release may contribute to the early stages of epileptogenesis, it is very possible that some proteins are more important in the maintenance of this epileptic state once an animal is fully kindled. This emphasizes the complexity of the molecular mechanisms involved in epileptogenesis, and accentuates the fact that these proteins and their association with neurotransmitter release need to be studied at many different time points in the epileptogenic process to fully understand the dynamic interactions that contribute to epileptogenesis.

We saw only minimal differences in the 7SC complex, SV2, or NSF in this study that did not reach statistical significance. This result was not as robust as our previous findings in several kindling models where we saw changes in these protein complexes in the hippocampi of both kindled rats and mice (Matveeva et al., 2007, 2011, 2012). In mice we observed minor, yet significant, increases in SV2 with minimal changes in NSF while observing more robust alterations in the 7SC (Matveeva et al., 2011). Ohno et al. (2009) also has reported a preferential increase in the hippocampal SV2A following pentylenetetrazole kindling. The lack of more robust changes in the present study may be related to strain background since these animals were not on a C57Bl/J background. Alternatively, the loss of STXBP5/tomosyn‐1 could affect the biochemical changes that we noted previously. However, to fully understand the mechanism(s) for the dampening of the protein and complex changes, more experiments are needed.

In summary, these data suggest Tom^−/−^ mice progress through kindling faster requiring fewer stimuli to reach a fully kindled state and that naïve Tom^−/−^ animals do have higher evoked glutamate release compared to naïve Tom^+/+^ mice before kindling occurs. Furthermore, after animals have reached two consecutive Racine stage 5 seizures, kindling increases the amplitude of KCl‐evoked and spontaneous glutamate release in the DG. These results show the benefit of directly measuring glutamate in kindled and transgenic animals to understand how glutamate dynamics are involved in kindling and epileptogenesis and highlight the complexity of protein–protein interactions in regulating neurotransmitter dynamics throughout the epileptogenic process.

Recent years have seen the emergence of the concept of “synaptopathies”, alterations in the neurotransmitter release machinery, as a cause of certain human epilepsies. These include mutations in syntaxin‐1B (STX1B), SNAP‐25, and syntaxin binding protein 1 (STXBP1/Munc 18‐1) (Rohena et al., 2013; Saitsu, Kato, Mizuguchi, Hamada, & Osaka, 2008; Schubert, Siekierska, Langlois, May, & Huneau, 2014). The current study of kindling facilitation in a viable STXBP5/tomosyn‐1‐deficient mouse suggests another potential synaptopathy that has not been described in humans.

## CONFLICT OF INTEREST

G. A. Gerhardt is the principal owner of Quanteon, LLC that makes the FAST 16 mKII recording system used in these studies. None of the other authors has any conflicts to disclose. We confirm that we have read the Journal's position on issues involved in ethical publication and affirm that this report is consistent with those guidelines.
